# Morphology of paraspinal muscles in frail and non-frail older adults evaluated through FRAIL scale

**DOI:** 10.1186/s12891-023-06144-z

**Published:** 2023-01-17

**Authors:** Rufeng Huang, Fumin Pan, Chao Kong, Shibao Lu

**Affiliations:** 1 Department of Orthopedics, Xuanwu Hospital, Capital Medical University, Beijing, China; 2 National Clinical Research Center for Geriatric Diseases, Beijing, China

**Keywords:** Paraspinal muscles, FRAIL, Elderly, Lumbar degenerative disease

## Abstract

**Background:**

Frailty is a condition characterized by the progressive deterioration of physiological functioning, which is closely related to adverse events. Multiple previous investigations applied frailty scales for spine research, and the purpose of this study is to investigate the differences in the morphology of the paraspinal muscles in frail and non-frail older adults evaluated through FRAIL scale.

**Methods:**

The sample of this retrospectively cross-sectional study consisted of individuals who were ≥ 60 years of age and with lumbar degenerative disease. We divided patients into two groups (0–2 = non-Frail, 3–5 = Frail) according to the FRAIL scale. The cross-sectional area (CSA) and percentage of the fatty infiltration (FI%) of the paraspinal muscles were compared between the two groups.

**Results:**

The fCSA (functional cross-sectional area) of the non-Frail group (32.78 [28.52, 38.28]) (cm^2^) was significantly greater than that of the Frail group (28.50 [24.11, 34.77]) (*p* < 0.001). The ES FI% (erector spinae fatty infiltration rate) (24.83 ± 6.61 vs. 29.60 ± 7.92, *p* < 0.001) and MF FI% (multifidus fatty infiltration rate) (31.68 ± 5.63 vs. 41.12 ± 7.04, *p* < 0.001) of the non-Frail group were significantly lower than that of Frail group.

**Conclusions:**

The paraspinal muscles of elderly Frail patients screened by the FRAIL scale are worse than those of the non-Frail patients, and the ability of the FRAIL scale to distinguish paraspinal muscle morphology has important clinical significance.

## Introduction

Frailty syndrome is a complicated age-related clinical disorder defined by biological vulnerability produced by a deterioration in functional reserve across various physiologic systems, decreased homeostatic capacity, and greater sensitivity to adverse events [1]. The increasing aging of the population has increased the number of older adults with frailty [2]. However, in multiple types of procedures, including spine surgery, increased frailty is associated with a greater incidence of postoperative adverse events and death [3]. Thus, effective screening solutions, preventing, and managing frailty in the aging population are likely to lessen the burden of the condition on both the person and the health system. Various frailty scales have been developed and used to screen frail patients [4]. In a recent systematic review [5], Kitamura et al. advocate, utilizing and further studying the FRAIL (fatigue, resistance, ambulation, illness, and loss of weight) scale for primary triage, which is a simple questionnaire (two domains, five items). Furthermore, this scale has previously been shown to predict the reduction in the activities of daily living and adverse events following elective spine surgery in research [6].

The paraspinal muscles, consisting of the multifidus muscle (MF) and erector spinae muscle (ES), play an important role in spinal movement and maintain stability [7]. An increasing number of studies have examined the correlation between the paraspinal muscles and spinal disease during the last decade, such as low back pain (LBP), lumbar spinal stenosis (LSS), sagittal imbalance and so on [7–9]. Fortin et al. proposed a correlation between paraspinal muscle morphology and functional status in patients with LSS [10]. Yoshida et al. found that larger preoperative paravertebral muscle mass were associated with improvement in sagittal imbalance following decompression surgery in LSS patients with sagittal imbalance [9]. Hence, it is imperative to assess the composition and function of the paraspinal muscles. Most studies employed magnetic resonance imaging (MRI) for muscle evaluation in the past, but the measurement process is cumbersome [11]. What’s more, the expense of MRI is significant, and some patients are still unable to undergo MRI due to contraindications or other issues. There have been multiple previous investigations applying frailty scales for spine research [12], while few of study on the application of FRAIL scales in the assessment of paraspinal muscles.

The purpose of this study is to investigate the differences in the morphology of the paraspinal muscles in frail and non-frail older adults evaluated through FRAIL scale.

## Material and method

### Patients

The ethical approval for this study was obtained from the Xuanwu hospital Ethics Commission (ID: 2,018,086). All the investigations were conducted in conformity with the applicable rules and the Declaration of Helsinki. Before enrolling and publishing identifiable information/images, all participants gave their informed consent. The sample of this retrospectively cross-sectional study consisted of individuals who were ≥ 60 years of age and with lumbar degenerative disease (LDD, including lumbar disc herniation, lumbar spinal stenosis, and degenerative spondylolisthesis). The patients with complete clinical and radiological data between January 2021 and January 2022 were retrospectively reviewed. Patients with lumbar fracture, scoliosis, neuromuscular illness, spinal cancer, or a history of spinal surgery were excluded from this study, and 200 patients were included finally [13]. The patient’s age, sex, and BMI are collected as demographic data for analysis.

### Assessment of Frailty

The FRAIL scale was published by Morley et al. and used to assess frailty [14]. The FRAIL scale consists of the following five items: fatigue, resistance, ambulation, illness, and loss of weight. (1) Patients were asked how exhausted they felt in the previous month to determine their level of fatigue. One point was given if they were exhausted all the time or most of the time. (2) Resistance was tested by asking whether it was difficult to ascend 10 stair steps without rest or assistance. If the response was yes, one point was given. (3) Ambulation was evaluated by questioning if it was hard to walk 300 m without additional help. If the response was yes, one point was given. (4) Illness was examined by inquiring if they had hypertension, diabetes, cancer, chronic lung disease, heart attack, congestive heart failure, angina, asthma, arthritis, stroke, or renal disease. It was given one point if they had five or more diseases at the time of the response. (5) Loss of weight was given one point if the patients had dropped more than 5% of their weight in the previous year. The response count was totaled, and patients were divided into two groups (0–2 = non-Frail, 3–5 = Frail) [15].

### Paraspinal muscles parameters

**Fig. 1:**
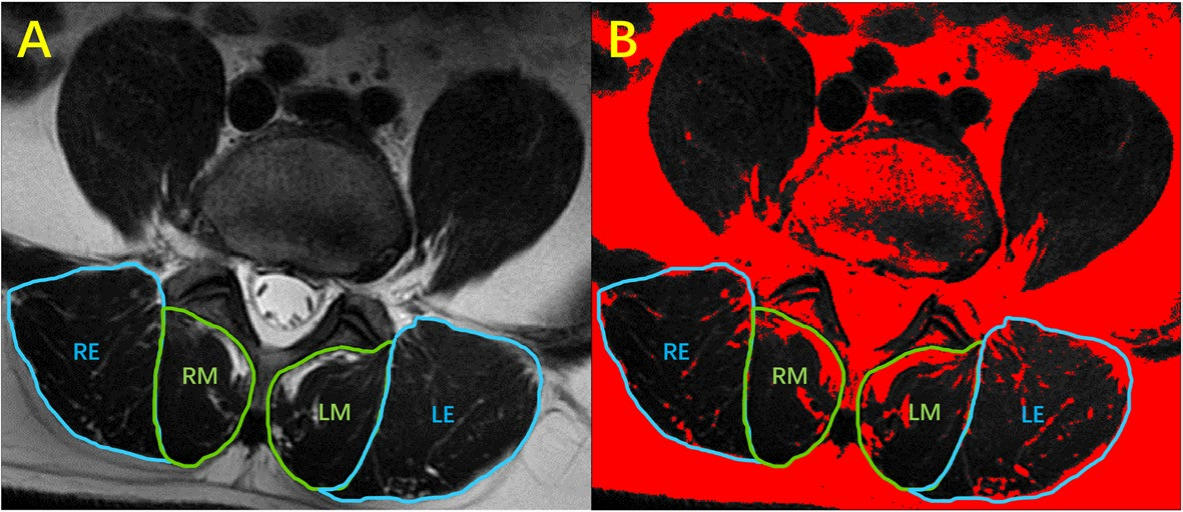
Fig. 1.**A** is a T2-weighted image of the axial L4-L5 disc level. RE was the CSA (cross-sectional area) of the right erector spinae, LE was the CSA of the left erector spinae, RM was the CSA of the right multifidus, LM was the CSA of the left multifidus. Figure 1.**B** is the image obtained from threshold processing of A by Image J. Fat (red area) and muscle tissue (without highlight area) were segmented, and the red area can be calculated by the software. tCSA = RE + RM + LE + LM, fCSA = tCSA-fat tissue (read area in four circles)

The cross-sectional area (CSA) and fatty infiltration (FI%) of the paraspinal muscles (erector spinae and multifidus) at the L4-L5 disc level were measured using the T2-weighted axial MRI by two skilled surgeons. They have been engaged in paraspinal muscle-related research for three years and were blind to the FRAIL scale data. The multifidus and erector spinae border were traced using the Image J image-processing software platform (National Institutes of Health, Bethesda, MD) [16]. The software will calculate the circle area according to the ruler entered in advance and take cm^2^ as the unit. According to the approach proposed by Fortin et al. [17], functional CSA (fCSA, lean paraspinal muscle) was calculated as total CSA (tCSA = RE + RM + LE + LM, Fig. 1.A) minus fat tissue area (red area in four circles, Fig. 1.B). The data were collected utilizing a very accurate thresholding approach based on the signal intensity difference between muscle and fat tissue. The fatty infiltration rate (FI%) was calculated by the ratio of the fat tissue area to the total cross-sectional area.

### Statistical analysis

The Statistical Package for the Social Sciences (SPSS, version 27.0, SPSS, Inc, Chicago, IL, USA) was used for all statistical analysis. Data normality was determined by the Shapiro–Wilk test. Continuous data with a normally distribution were represented as mean and standard deviation, while continuous variables with a non-normal distribution were expressed as median (1st quartile, 3rd quartile). Chi-square and Fisher's exact tests were used to assess categorical variables. The student's t-test and the Mann–Whitney U test were used to compare continuous variables. Multivariable regression was used to analyze the association between age, sex, BMI, frailty (all the patients, n = 200), and paraspinal muscle morphology (including fCSA and FI%). Logistic regression was used to analyze the association between fCSA, ES FI%, MF FI%, and frailty. Statistically significant difference was defined as a P value less than 0.05.

## Results

A total of 200 patients (non-Frail 127, Frail 73) were enrolled (Table 1). There was no significant difference between non-Frail and Frail groups in age, sex, and BMI. Among paraspinal muscle-related parameters, the fCSA of the non-Frail group was significantly greater than that of the Frail group, and the difference in tCSA was not significant. The ES FI% and MF FI% of the non-Frail group were significantly lower than that of the Frail group.

**Table 1:** Comparison of demographic and paraspinal muscle parameters between the non-Frail and Frail groups

	non-Frail (*n* = 127)	Frail (*n* = 73)	*P* value
Age	68 (64, 73)	70 (66, 73)	0.076
Male (%)	59 (46.46)	31 (42.47)	0.585
BMI	25.01 (22.86, 27.04)	25.24 (23.13, 28.18)	0.423
tCSA	46.42 ± 8.47	44.60 ± 8.27	0.143
fCSA	32.78 (28.52, 38.28)	28.50 (24.11, 34.77)	< 0.001*
ES FI%	24.83 ± 6.61	29.60 ± 7.92	< 0.001*
MF FI%	31.68 ± 5.63	41.12 ± 7.04	< 0.001*

*BMI* Body mass index, *tCSA* Total cross-sectional area, *fCSA* Functional cross-sectional area, *ES FI*% Erector spinae fatty infiltration rate, *MF FI*% Multifidus fatty infiltration rate, * Statistical significance at the level of 0.05

Table 2 is a multivariable regression analysis of the relationship between demographic parameters (age, sex, BMI), frailty, and fCSA. The results of multivariable regression analysis showed that the regression equation was significant. (F = 43.335, *p* < 0.001, adjusted R^2^ = 0.460). Age, sex, BMI, and frailty can significantly affect fCSA (*p* < 0.001). The standardized coefficients β of age, sex, BMI, and frailty were -0.209, -0.564, 0.224, and -0.241 respectively.

**Table 2:** Multivariable regression analysis of age, sex, BMI, frailty and fCSA

	B	95% CI for B	β	*p* value	VIF	Adjusted R^2^
Age	-0.267	(-0.260, -0.275)	-0.209	< 0.001*	1.015	0.460
Sex	-8.392	(-0.562, -0.611)	-0.564	< 0.001*	1.009	
BMI	0.444	(0.158, 0.292)	0.224	< 0.001*	1.016	
Frailty	-3.705	(-0.267, -0.311)	-0.241	< 0.001*	1.023	

*BMI* Body mass index, *VIF* Variance inflation factor, * Statistical significance at the level of 0.05

Table 3 is a multivariable regression analysis of the relationship between demographic parameters (age, sex, BMI), frailty, and FI%. The results of multivariable regression analysis showed that the regression equation was significant. (F = 35.371, *p* < 0.001, adjusted R2 = 0.409). Age, sex, and frailty can significantly affect fCSA (*p* < 0.001). The standardized coefficients β of age, sex, and frailty were 0.267, 0.332, and 0.415 respectively.

**Table 3:** Multivariable regression analysis of age, sex, BMI, frailty and FI%

	B	95% CI for B	β	*p* value	VIF	Adjusted R^2^
Age	0.003	(0.002, 0.004)	0.267	< 0.001*	1.015	0.409
Sex	0.044	(0.029, 0.058)	0.332	< 0.001*	1.009	
BMI	0.002	(0.000, 0.004)	0.105	0.058	1.016	
Frailty	0.056	(0.042, 0.071)	0.415	< 0.001*	1.023	

*BMI* Body mass index, *VIF* Variance inflation factor, * Statistical significance at the level of 0.05

Table 4 is a logistic regression analysis of the relationship between fCSA, ES FI%, MF FI%, and frailty. The goodness-of-fit of logistic regression analysis was better (Chi-square = 11.387, *p* = 0.181), and only MF FI% (OR = 1.267, *p *< 0.001) had a significant statistical difference.

**Table 4:** Logistic regression analysis of fCSA, ES FI%, MF FI%, and frailty

	B	Wald	*p* value	OR	95% CI for OR
fCSA	0.006	0.031	0.859	1.006	(0.943, 1.073)
ES FI%	0.009	0.084	0.772	1.009	(0.948, 1.074)
MF FI%,	0.237	39.040	< 0.001*	1.267	(1.176, 1.364)

*fCSA* Functional cross-sectional aera, *ES FI*% Erector spinae fatty infiltration rate, *MF FI*% Multifidus fatty infiltration rate, * Statistical significance at the level of 0.05

## Discussion

We evaluated patients with LDD using the FRAIL scale and discovered that the Frail patients had considerably poor lumbar paraspinal muscle morphology and more significant fatty infiltration than the non-Frail patients. There doesn’t appear to be any past research similar to this study.

Paraspinal muscles play an essential role in maintaining the normal shape of the spine [18], and its composition is closely related to spinal diseases, including LBP, lumbar disc herniation (LDH), LSS, spondylolisthesis and spinal imbalance, and the prognosis of spinal surgery [19]. Fortin et al. found that multifidus muscle FI and psoas relative CSA are associated with functional status in patients with LSS [10]. A systematic review conducted by Jermy et al. confirmed an association between low multifidus fat infiltration on MRI at baseline and greater reductions in measures of LBP and disability following surgical treatment [20]. Tang et al. indicated that patients with degenerative lumbar scoliosis had asymmetric degeneration of the paraspinal muscles and psoas major. The CSA and FI of the multifidus are closely correlated with the quality of life [21]. Some LSS and coexisted with sagittal imbalanced patients were reported to restore their normal sagittal morphology only through simple decompression or limited decompression and fusion surgery [9, 22, 23]. However, these studies have shown that patients with relatively stronger paraspinal muscle mass could restore normal spine alignment after surgery.

Through the regression analysis, we found that besides sex, frailty has the greatest influence on fCSA among these factors, and it is a risk factor for fCSA of the paraspinal muscles. Frailty also has the most significant impact on FI% of the paraspinal muscles among these factors. In other words, frail patients would have smaller fCSA, and a greater fatty infiltration rate in paraspinal muscles, which means a worse paraspinal muscle morphology. From the regression analysis, it can be found that only the FI% of MF has a significant effect on frailty, which means that the patients with greater MF FI% would be more likely to suffer from frailty. Therefore, frailty and morphology of paraspinal muscle would affect each other, frailty will worsen the paraspinal muscle’s morphology, and poor paraspinal muscle morphology is a risk factor for frailty. This suggests that strengthening paraspinal muscle exercise would be helpful to improve the frail status of patients. Moreover, considering the critical role of paraspinal muscles in maintaining the curve of the spine and its close relationship with lumbar degenerative diseases, it is also vital to remedy the frail status in LDD therapy. Therefore, assessment of the muscle mass presents important clinical implications before developing a treatment plan. In this study, only asked a few simple questions and could make a preliminary judgment on the condition of a patient's paraspinal muscles. It is self-evident that it brings convenience to surgeons and low cost to patients in clinical work.

Frailty is a condition characterized by the progressive deterioration of physiological functioning, which is closely related to adverse events such as falls, decreasing mobility, hindrances in the activities of daily life, hospitalization, and deaths [24]. Frailty leads to physical deterioration and reduced mobility. Therefore, lacking exercise and motion, the process of muscle degeneration in frail patients would be more severe than in those not [25]. Martino et al. found that intramuscular lipid concentration increased in localized regions of the lumbar muscles following 60-day bed rest [26]. Previous studies have shown nutritional risk factors were independently associated with physical pre-frail/frail condition [27]. However, poorer nutritional status is significantly associated with sarcopenia [28]. As a result, it also explains why the paraspinal muscles of Frail patients are worse than non-Frail patients.

The FRAIL scale was published by Morley et al. and used to assess frailty, which has been demonstrated as an excellent screening test for clinicians to identify frail persons at risk of developing disability as well as a decline in health functioning and mortality [14, 24]. Although MRI is more accurate in assessing paraspinal muscle mass, it has the disadvantage of being time-consuming, labor-intensive, and expensive. Additional extensive, multi-dimensional frailty screening scales and prospective study protocols could be utilized in future investigations to determine the validity and reliability of this conclusion.

There are still some limitations that should be considered. First, the characteristics of the research cohort cannot be typical of the general population because we only included relatively small Chinese senior patients. Second, this is only a retrospective study, and other prospective results are needed to support the clinical relevance and implications. Finally, the current study used a basic frailty screening tool to evaluate the influence of the frailty state on the paraspinal muscle’s morphology. Compared with other complex or multidimensional scales, the FRAIL scale lacks a certain amount of relative accuracy. However, its convenience could save a lot of time and boost the efficiency of clinicians.

## Conclusion

The paraspinal muscles of elderly Frail patients screened by the FRAIL scale are worse than those of non-Frail patients, and the ability of the FRAIL scale to distinguish paraspinal muscle morphology has important clinical significance.

## Data Availability

The datasets generated and/or analyzed during the current study are not publicly available due to data privacy rules but are available from the corresponding author on reasonable request.
